# Fibroblast growth factor 1 ameliorates thin endometrium in rats through activation of the autophagic pathway

**DOI:** 10.3389/fphar.2023.1143096

**Published:** 2023-04-20

**Authors:** Jing Zhu, Zhenyao Li, Fengli Yin, Xiaoting Yu, Yuanfan Lu, Tong Zhou, Fanghua Gong, Zhangye Xu

**Affiliations:** ^1^ Department of Gynecology and Obstetrics, The Second Affiliated Hospital and Yuying Children’s Hospital of Wenzhou Medical University, Wenzhou, China; ^2^ School of Pharmacy, Wenzhou Medical University, Wenzhou, China

**Keywords:** thin endometrium, uterus damage, fibroblast growth factor 1, reproductive capacity, intrauterine insemination

## Abstract

**Background**: Thin endometrium is a reproductive disorder that affects embryo implantation. There are several therapies available for this disease, however they are not so effective. Fibroblast growth factor 1 (FGF1) is a member of fibroblast growth factor superfamily (FGFs), and it has been demonstrated that FGF1 expression was altered in samples collected from patients with thin endometrium. However, it is unclear if FGF1 could improve thin endometrium. The aim of this study was to investigate whether FGF1 have a therapeutic effect on thin endometrium.

**Methods:** A model of thin endometrium induced by ethanol was constructed to investigate the effect and mechanism of action of FGF1 in thin endometrium. In the characterization experiments, 6–8 weeks female rats (n = 40) were divided into four groups: i) Control group; ii) Sham group; iii) Injured group; (iv) FGF1 therapy group. Endometrial tissues would be removed after three sexuel cycles after molding. Morphology and histology of the endometrium were evaluated by visual and hematoxylin and eosin staining. Masson staining and expression of α-SMA in endometrium showed the degree of endometrial fibrosis. Western blotting (PCNA、vWF and Vim) and immunohistochemistry (CK19 and MUC-1) demonstrated the effect of FGF1 on cell proliferation and angiogenesis. Moreover, immunohistochemistry (ER and PR) was used to explore the function of endometrium. The remaining rats (n = 36) were divided into three groups: i) Injured group; ii) FGF1 therapy group; and iii) 3-methyladenine. Western blotting (p38、p-p38、PI3K 、SQSTM1/p62、beclin-1 and LC3) was used to explore the mechanisms of FGF1.

**Results:** In FGF1 therapy group, the morphology and histology of endometrium improved compared with the model group. Masson staining and the expression level of α-SMA showed that FGF1 could decrease the fibrotic area of endometrium. Besides, changes in ER and PR expression in the endometrium suggested that FGF1 could restore endometrium-related functions. Western blotting and immunohistochemistry revealed that PCNA, vWF, Vim, CK19 and MUC-1 were significantly increased after FGF1 treatment compared with the thin endometrium. In addition, Western blotting showed that p38, p-p38, PI3K, SQSTM1/p62, beclin-1 and LC3 levels were higher in FGF1 group than in the injured group.

**Conclusion:** FGF1 application cured the thin endometrium caused by ethanol through autophagy mechanism.

## Background

The endometrium is an important part of the female productive system, which comprises glands, vessels, epithelium and stroma. Previous studies have demonstrated that damage and changes in the tissue composition of the uterus can have a marked impact on the normal physiological cycle, embryonic implantation and pregnancy (Critchley et al., 2020; Ganieva et al., 2022; Goharitaban et al., 2022). Furthermore, the thickness of the endometrium has an effect on the process of embryonic embedding, delivery and birth weight of the fetus (Moffat et al., 2017; Zhang et al., 2019; Guo et al., 2020; Liu et al., 2021; Lin et al., 2022). In recent studies, changes in endometrial thicknesses had a substantial effect on embryo survival after intrauterine insemination, and post-operative embryo survival rates decreased as the recipient woman’s endometrial thickness decreased (Liu et al., 2018; Mahutte et al., 2022).

Over the years, a number of hypotheses have been proposed regarding the pathogenesis of thin endometrium, including increased cellular senescence, excessive collagen deposition and downregulation of gene expression, based on thin uterine disease models (Ziganshina et al., 2021; Lv et al., 2022). Therefore, a variety of therapeutically suggested therapies have been created to target these pathogenic pathways. For example, results of vaginal sildenafil therapy in patients with clinical failure of *in vitro* fertilization due to endometrial dysplasia have been examined (Barad et al., 2014). Extended estrogen, low-dose aspirin, granulocyte colony-stimulating factor intrauterine infusion therapy and other therapies have been available in addition to the two treatments already mentioned (Li et al., 2014; Gharibeh et al., 2022). However, occasionally, the efficacy of these treatment techniques is questionable. Therefore, the present study aimed to determine if there are any more elements that could be used to treat thin endometrium.

Fibroblast growth factor 1 (FGF1), also known as acid FGF, which is involved in tissue regeneration, trauma repair and metabolism (Hwang et al., 2022; Li et al., 2022; Qin et al., 2022; Sancar et al., 2022). However, in recent years, numerous studies have indicated that FGF1 also appeared to have an effect on the endometrium (Okumu et al., 2014; Kato et al., 2019). Additionally, one past study found that FGF1 expression levels were higher in patients with successful implantation than patients with repeated implantation failure in the important role of the fertilization process *in vitro* (Sak et al., 2013). FGF receptor 1 is most strongly expressed during endometrial secretion, consistent with the development of endometrial edema and the formation of complex capillary plexus, and is critical for endometrial angiogenesis, re-epithelialization, vascular function and stromal cell proliferation (Gao et al., 2019).

In the present study, a rat model of thin endometrium was utilized, and rats were treated with FGF1 to investigate the effect of FGF1 on thin endometrium and its possible mechanism.

## Methods

### Animals

76 healthy female Sprague Dawley rats (weight, 250–280 g; age, 6–8 weeks) purchased from Charles River Laboratories, Inc. were used. At the Animal Experiment Center of Wenzhou Medical University, all rats were kept in cages in designated microbe rooms at room temperature, with a humidity level of ∼60% and a 12/12-h light period cycle. Before the tests, the animals were given a week to acclimate.

The vaginal secretions of the rats were collected every day at 8:00 a.m. after 1 week of acclimation upbringing. Based on the 7-day vaginal smear, the sexuel cycle of the rats could be determined, and the rats who were in the estrus were selected to construct the lamellar uterine model.

All animal experiments were performed in compliance with guidelines and approved by the animal ethics committee of Wenzhou Medical University, approval no. xmsq 2022–1166.

### Ethanol-induced thin endometrium modeling

The female rats were anesthetized by intraperitoneal injection of 1% pentobarbital sodium (40 mg/kg) before the abdominal cavity was opened to reveal the uterus. Subsequently, the uterine horns were separated and the ovarian and vaginal ends of the uterus were tied with surgical thread. Thereafter, 95% ethanol was slowly injected in each uterine horn, until the uterus was full. After 5 min, the ethanol was sucked up using an injector. The intrauterine cavity was flushed with saline and the abdomen was closed with stitches. The rats were placed in a warm environment to awaken. All rats were euthanized by 70% vol/min CO_2_ asphyxiation after three erogenous cycles, and the uterine tissue was removed.

While the remaining uterine horns were cut open and the endometrium was taken for preservation in liquid nitrogen for further tests, a part of the retrieved uterine horns was fixed (70% ethanol overnight, 80% ethanol 1 h, 95% ethanol 30 min, 95% ethanol 30 min, 100% ethanol 15 min, 100% ethanol 15 min, xylene 15 min, xylene 15 min) and imbedded immediately in paraffin.

### Animal grouping and treatment

In the characterization experiments, the rats (n = 40) were divided into four groups on average: i) Control group; ii) Sham group; and iii) Injured group; (iv) FGF1 therapy group. The rats in the control group underwent no procedures. In the sham group, saline was instilled into the uterine cavity until filled and then aspirated after opening the abdomen. After that sutured the rats until they awoke. In the therapy group, FGF1 (1 mg/kg) was administered to the intrauterine after 95% ethanol damage. In order to verify the mechanism of the effect of FGF1, the remaining rats (n = 36) were separated into three groups on average: i) Injured group; ii) FGF1 therapy group; and iii) 3-methyladenine (3-MA; M129496; Shanghai Aladdin Biochemical Technology Co., Ltd.) group. The first two groups were treated as before, and the rats in the 3-MA group were injected intraperitoneally with 3-MA (10 mg/kg) for 1 week after administration of FGF1.

### Hematoxylin-eosin (H&E) and Masson staining

Paraffin-embedded sections (4 μm) were deparaffinized with xylene (15 min; twice) and rehydrated with decreasing ethanol concentrations (100, 100, 90, 80% and 70%; 5 min each) and pure water. After H&E staining (G1120; Beijing Solarbio Science & Technology Co., Ltd.) and Masson staining (G1340; Beijing Solarbio Science & Technology Co., Ltd.), the slides were dehydrated with an ascending concentration of alcohol and clarified by xylene. Lastly, the slides were blocked with neutral resin and viewed under a microscope and camera.

A total of three portions of a uterine specimen were randomly selected for observation of endometrial thickness, number of endometrial glands and amount of blood vessels. Endometrial thickness was calculated vertically at 3, 6, 9o and 12o’clock on each section. The average of the four values was calculated for each slice and considered to be the normal endometrial layer thickness of the slice. The mean of the three measurements was then used as the final endometrial thickness. In addition to this, the average numbers of glands and blood vessels of three different samples were calculated.

Masson staining was carried out as described in the manual. The fibrotic areas were colored light blue and assessed using ImageJ 1.50i software (National Institutes of Health). All sample selections were based on the principle of randomness.

### Immunohistochemistry staining

After dewaxing, tissue sections were soaked in sodium citrate buffer, placed in an autoclave for antigenic thermal repair and pretreated with 0.3% H_2_O_2_ in methanol to quench the endogenous peroxidase activity. Subsequently, the sample was incubated with 5% bovine serum albumin (BSA) at 37°C 30 min for blocking. Primary antibodies, including anti-α-smooth muscle actin (α-SMA; 1:1,000; 80008-1-RR; Proteintech Group, Inc.), anti-mucin 1 (MUC-1; 1:100; MA5-35250; Thermo Fisher Scientific, Inc.), anti-estrogen receptor (ER; 1:100; 21244-1-AP; Proteintech Group, Inc.), anti-progesterone receptor (PR; 1:100; sc-810; Santa Cruz Biotechnology, Inc.) and anti-cytokeratin 19 (CK19; 1:200; 10712-1-AP; Proteintech Group, Inc.), were used to incubate the tissue overnight at 4°C. After the primary antibody was fully reacted, the secondary antibody was applied and the subsequent steps were carried out according to the instructions of the immunohistochemistry kit (KIT-9710; Fuzhou Maixin Biotech Co. Ltd.), followed by 3,3′-diaminobenzidine development and hematoxylin staining for differentiation, and microscopic observation of the blocked slices.

### Western blot analysis

Protein in the rat endometrium was obtained through tissue lysate to get a histone stock solution, with subsequent determination of the total protein content of the stock solution using a BCA protein assay kit to produce a Western blotting sample. A series of samples were loaded into a SDS polyacrylamide gel and transferred to a PVDF membrane. Membranes were incubated with anti-proliferating cell nuclear antigen (PCNA; 1:1,000; 10205-2-AP; Proteintech Group, Inc.), anti-vimentin (vim; 1:1,000; 10366-1-AP; Proteintech Group, Inc.), anti-von Willebrand factor (vWF; 1:1,000; 11778-1-AP; Proteintech Group, Inc.), anti-β-actin (1:3,000; 81115-1-RR; Proteintech Group, Inc.), anti-phosphoinositide 3-kinase (PI3K; 1:1,000; 20584-1-AP; Proteintech Group, Inc.), anti-p38 (1:1,000; 14064-1-AP; Proteintech Group, Inc.), anti-phosphorylated p38 (p-p38; 1:1,000; 28796-1-AP; Proteintech Group, Inc.), anti-sequestosome 1/p62 (SQSTM1/p62; 1:1,000; ab56416; Abcam.), anti-beclin-1 (1:5,000; 11306-1-AP; Proteintech Group, Inc.) and anti-MAP1LC3 (LC3; 1:5,000; #2775; Cell Signaling Technology, Inc.) antibodies at 4°C on a shaker overnight. On the second day, the PVDF membranes were incubated with anti-rabbit (1:20,000; 511203; Chengdu Zen-Bioscience Co., Ltd.) and anti-mouse (1:20,000; 511103; Chengdu Zen-Bioscience Co., Ltd.) secondary antibodies for up to 1 h at room temperature. The protein bands were visualized and analyzed using a Bio-Rad Chemi Doc XRS + Imaging System. The grayscale of the images was analyzed using ImageJ (National Institutes of Health).

### Statistical analysis

GraphPad Prism software (GraphPad Software, USA) was used to determine the differences among groups. All data are presented as the mean ± standard error of the mean. An unpaired two-way ANOVA (p-p38, SQSTM1/p62 and LC3-II in the experimenting with mechanism exploration) and Student’s t-test (remaining experimental indicators) were utilized for inter-group comparisons. *p <* 0.05 was considered to indicate a statistically significant difference.

## Results

### FGF1 promotes the morphological and histological changes of endometrium repair

**FIGURE 1:**
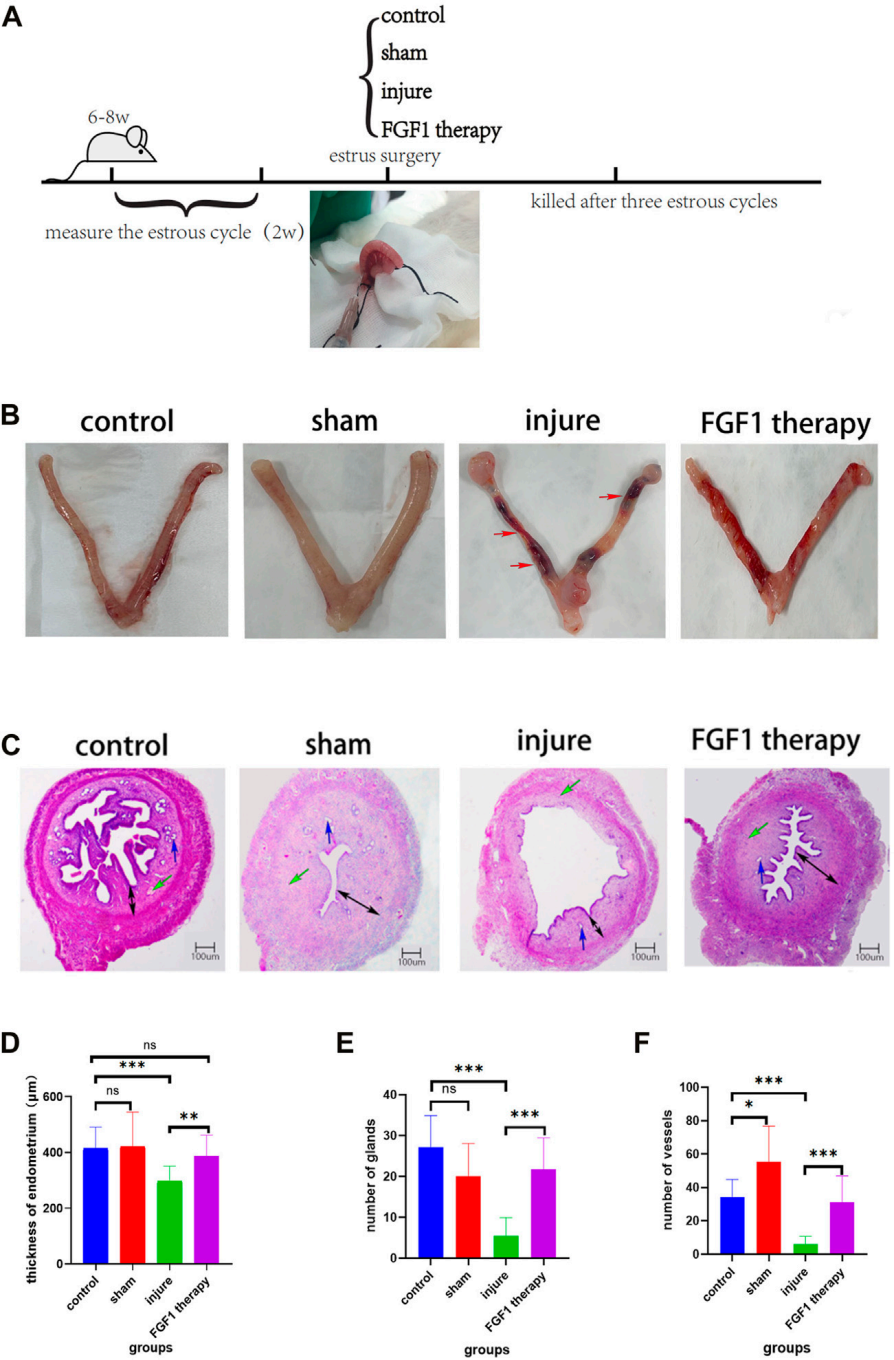
Morphological and histological structure of endometrium. Thin endometrial construction and experimental groups **(A)**; The morphology of the uterus in different groups. The red arrow points to the area of uterine stenosis and bruising following ethanol injury **(B)**; Histological features of the endometrium after HE staining. The blue arrow points to the endometrial glands. The green arrow points to endothelial vessels. The black arrow represents the thickness of the endometrium. HE, 40X **(C)**; Changes in endothelial thickness between groups. n = 10, ns > 0.05, **p < 0.01, ***p < 0.001 **(D)**; Changes in the number of endometrial glands between groups. n = 8, ns > 0.05, ***p < 0.001 **(E)**; Changes in the number of endometrial vessels between groups. n = 10, *p < 0.05, ***p < 0.001 **(F)**.

Rats were sacrificed by 70% vol/min CO_2_ asphyxiation three estrus cycles after the establishment of thin endometrium (Figure 1A). Uterine morphology and endometrial changes were the most obvious features of a thin uterus. In the control and sham groups, the uterine tissue was normal in appearance, red and full of elasticity. By contrast, in the injured group, there was obstruction, stenosis and even fluid accumulation in the tissues of the uterus. Following FGF1 treatment, the oedema and bruising brought on by ethanol damage generally subsided, and the uterus became red and glossy (Figure 1B). These indications suggested that FGF1 treatment had a restorative effect on the morphological appearance of the damaged tissues. The endometrium tissues were examined using H&E staining (Figure 1C). When rat endometrial samples were stained with H&E, alterations in the thickness of the endometrium were observed. As seen in the diagram (Figure 1D), the endometrium of the injured group was noticeably thinner than that of the control group (*p <* 0.001). However, FGF1 treatment increased the thickness compared with the injured group (*p <* 0.01). The quantity of blood vessels and endothelial glands was seen in a similar manner at the same time (Figures 1E, F). After injury, the number of glands and blood vessels in the endometrium was significantly reduced (*p <* 0.001), whereas in the FGF1 treated group the amount of blood vessels and glands in the endometrium was seen to be greatly increased (*p <* 0.001). The results of the aforementioned experiments demonstrated that intrauterine treatment with FGF1 increased the thickness of the thin endometrium caused by the injury and aided the regeneration of the endometrial glands and vasculature.

### FGF1 decreases the degree of fibrosis in thin endometrium

**FIGURE 2:**
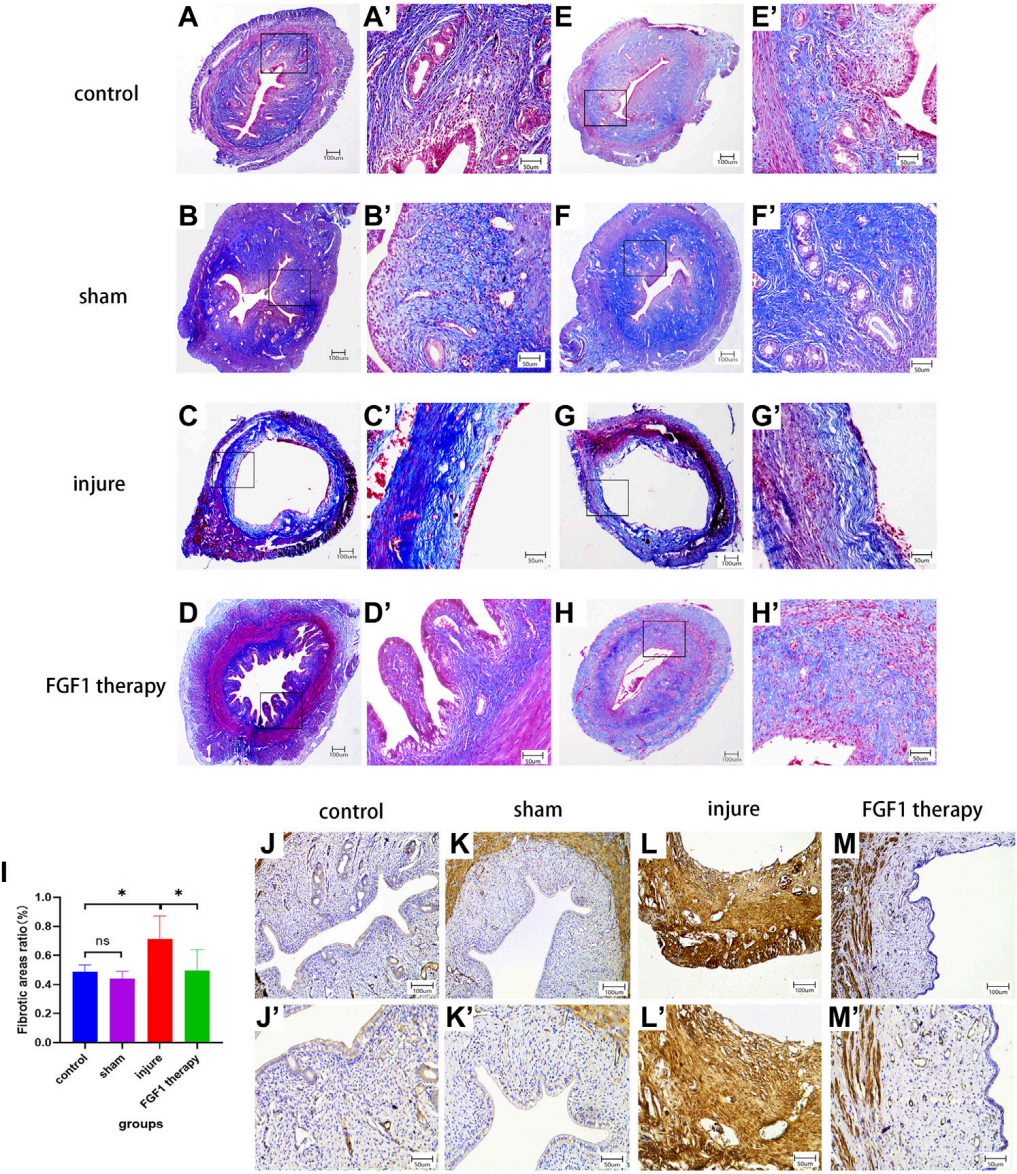
FGF1 reduces the degree of thin endometrial fibrosis. Masson staining of the endometrium. **(A–H)** 40X, **(A’–H’)** 200X. Fibrotic area to endothelial area ratio statistics by each group. n = 6, ns > 0.05, *p < 0.05 **(I)**. IHC results shows variations in αSMA expression in each experimental team. **(J–M)** 100X, **(J’–M’)** 200X.

One of the characteristics of a thin endometrium is endometrial fibrosis (Zhang et al., 2022). After Masson staining, the fibers in the endometrial tissue were dyed blue, and this was used to examine the therapeutic impact of FGF1 on the management of thin endometrial fibrosis (Figures 2A–H, 2A′-H′). The fibrous ratio of endometrial tissue in the injured group was higher than that in the control group (*p <* 0.05). However, FGF1 therapy markedly reduced intrauterine fibrosis (*p <* 0.05; Figure 2I). α-SMA is a myofibroblast marker that indicates the level of fibrosis. Immunohistochemistry revealed that the endometrium of the model group was brown, indicating marked α-SMA expression and endometrial fibrosis. However, the endometrium of the FGF1 therapy group did not exhibit any significant α-SMA-positive staining, suggesting that FGF1 reduced the development of endometrial fibrosis and had a greater therapeutic influence on thin endometrium (Figures 2J–M, 2J′–M′).

### FGF1 increases cell proliferation and angiogenesis in the endometrium

**FIGURE 3:**
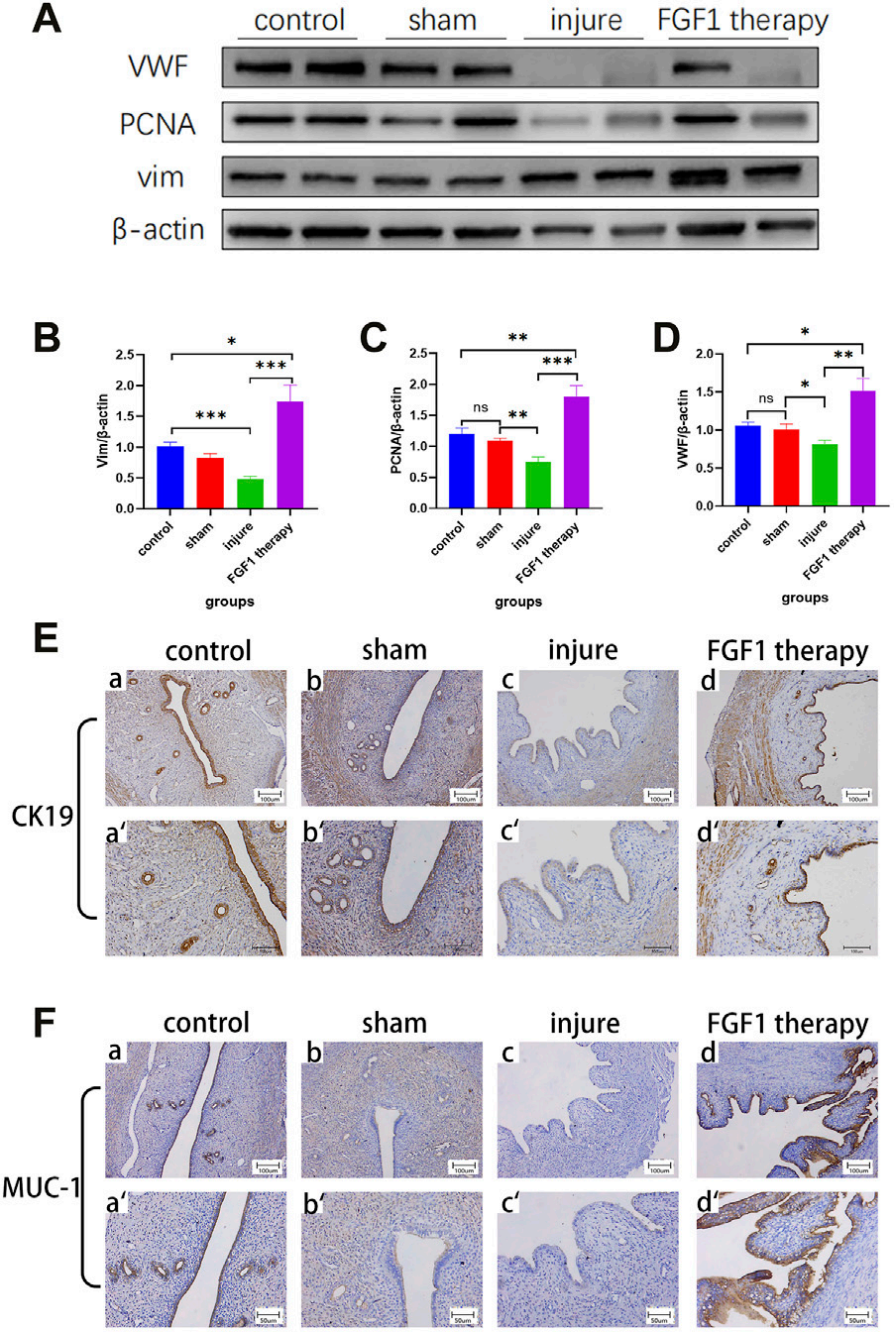
FGF1 promotes the expression of endothelial cell proliferation and angiogenic markers. The results of PCNA, vim, vWF and corresponding total protein Western blot **(A)**. Statistical analysis of PCNA, vim and vWF Western blot outcomes. n = 7, ns > 0.05, *p < 0.05, **p < 0.01, ***p < 0.001 **(B**–**D)**. Expressions of CK19 and MUC-1 in glandular epithelial and luminal epithelium cells of endometrium with IHC staining. a-d 100X, a’–d’ 200X **(E**, **F)**.

The present study revealed that the injured uterine lining exhibited a marked amount of fibrous collagen along with a fluctuation in the number of cells and blood vessels based on H&E staining. Western blotting was performed to examine the vascular hyperplasia protein indicator vWF, the cell proliferation protein indicators PCNA and vim, and the injury-induced alterations in cells and vessels to evaluate specific changes in cells and vessels (Figure 3A). Regarding PCNA (Figure 3C), the results demonstrated that it was markedly less expressed in the endometrium of the injured group than in that of the sham group (*p <* 0.01). On the other hand, the FGF1-treated group exhibited increased expression of this protein (*p <* 0.001). The same pattern was also observed for vim and vWF (Figures 3B, D). In addition to the previous markers examined, immunohistochemistry was used to determine the amounts of CK19 and MUC-1 indicator expression in the endometrium within each subgroup. The expression levels of CK19 and MUC-1 were noticeably reduced in the endometrium of the injured group, as shown in the figure (Figures 3E, F). After FGF1 perfusion, the expression levels of these two proteins were increased.

### FGF1 has a restorative effect on the function of thin endometrium

**FIGURE 4:**
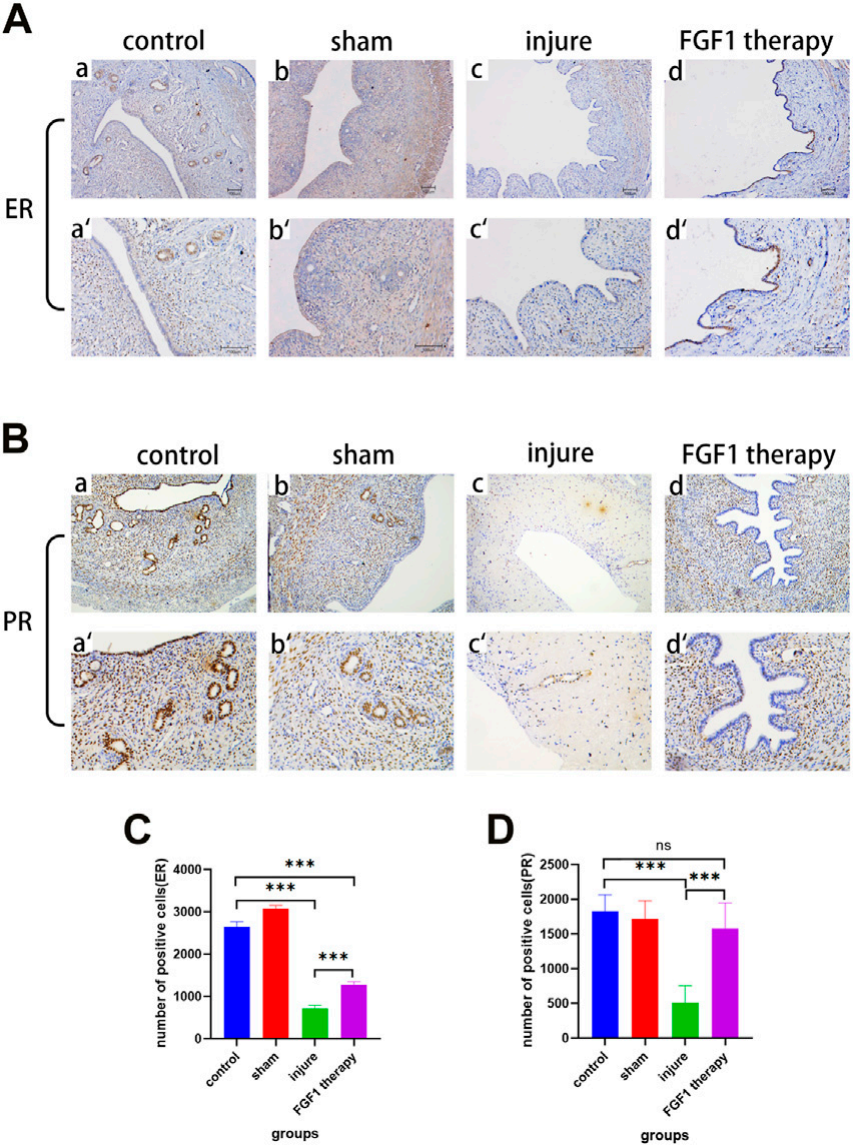
FGF1 increases ER and PR expression in the endometrium and restores uterine function. The results of ER expression by using IHC. a-d 100X, a’–d’ 200X **(A)**. The results of PR expression by using IHC. a-d 100X, a’–d’ 200X **(B)**. Analysis of ER IHC results, the number of positive cells expressing ER. n = 7, ***p < 0.001 **(C)**. Analysis of PR IHC results, the number of positive cells expressing PR. n = 6, ns > 0.05, ***p < 0.001 **(D)**.

ER and PR are two key protein indicators of endometrial function. In the past, it has been demonstrated that endometrial thinning was associated with abnormal expression of ER, while PR expression was not significantly different (Gao et al., 2019). Therefore, immunohistochemistry was used to examine the changes in ER and PR expression in the endometrium (Figures 4A, B). The experimental results clearly demonstrated that ER expression was decreased after endothelial injury (*p <* 0.001), which was consistent with the previous finding. Additionally, this reduction was somewhat improved after FGF1 treatment (Figure 4C; *p <* 0.001). However, the results for PR deviated from the previous result, as the change in PR expression in the present experiments was consistent with that of ER (Figure 4D).

### Recovery of thin endometrium by FGF1 *via* the autophagic pathway

**FIGURE 5:**
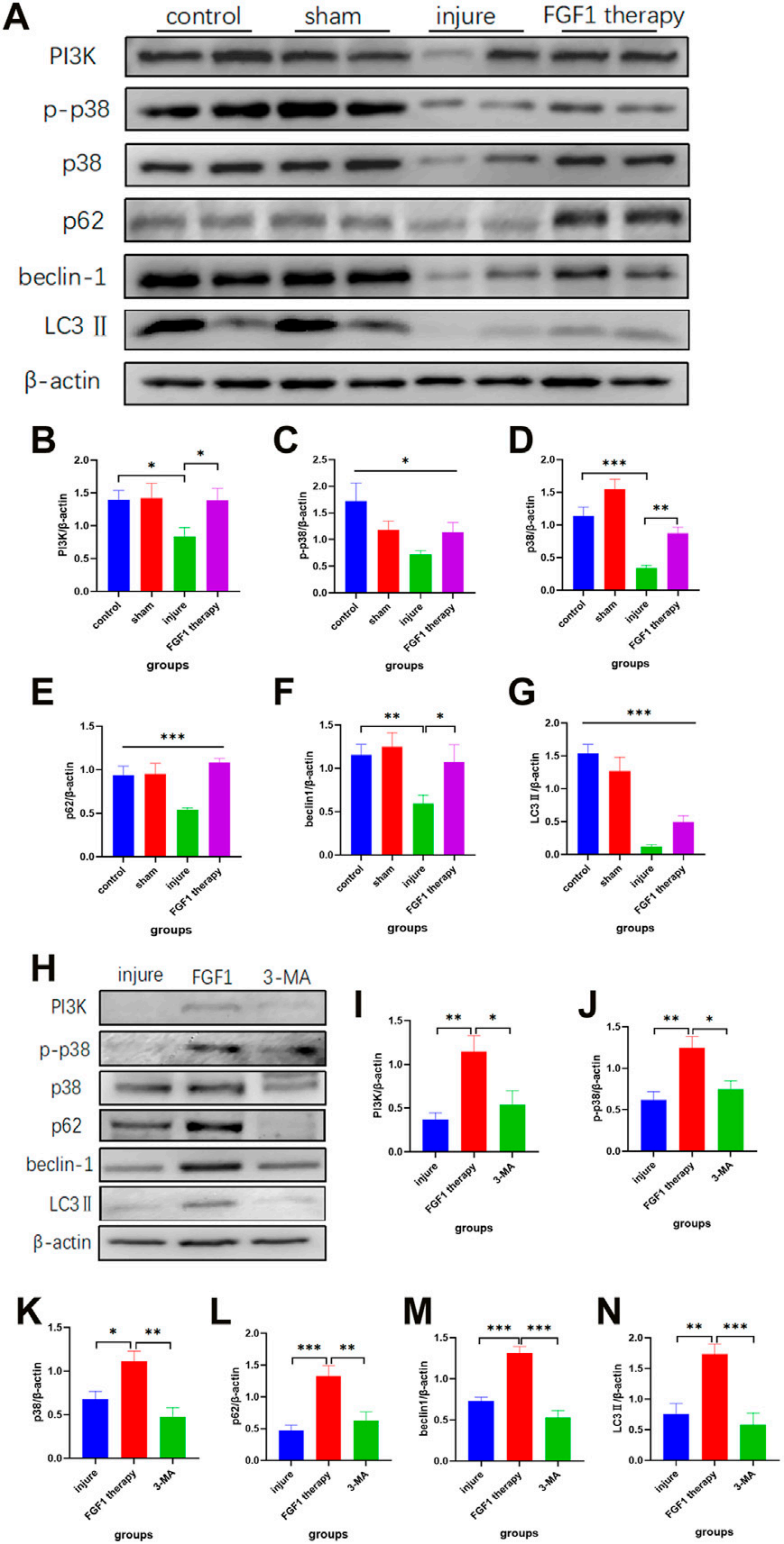
Endometrial repair by FGF1 is achieved mainly through activation of the PI3K/p38/Beclin 1 pathway. Trends in the expression of PI3K, p-p38, p38, p62/SQSTM1, beclin1 and LC3Ⅱ indicator proteins after FGF1 treatment revealed by Western blot experiments **(A)**. Statistical analysis of the expression with PI3K (n = 6) **(B)**, p-p38 (n = 7) **(C)**, p38 (n = 6) **(D)**, p62/SQSTM1 (n = 8) **(E)**, beclin1 (n = 8) **(F)** and LC3Ⅱ (n = 8) **(G)** in Western blot. *p < 0.05, **p < 0.01, ***p < 0.001. Intraperitoneal injection of 3-MA blocks protein expression of related pathways after activation of the autophagic pathway **(H)**. Statistical analysis of the expression with PI3K(n = 6) **(I)**, p-p38 (n = 8) **(J)**, p38 (n = 7) **(K)**, p62/SQSTM1(n = 6) **(L)**, beclin1 (n = 12) **(M)** and LC3Ⅱ (n = 8) **(N)** in Western blot after injecting 3-MA. *p < 0.05, **p < 0.01, ***p < 0.001.

The autophagic pathway serves a variety of roles in the sustenance and evolution of a number of tissues, including endometrial tissue (Mestre Citrinovitz et al., 2019). Additionally, FGF1 has been found to be associated with the autophagic pathway in the previous study (Li et al., 2018). Therefore, we hypothesized that FGF1 might promote endothelial recovery by activating the autophagic pathway. To confirm our hypothesis, the expression levels of proteins related to the autophagic pathway were examined by Western blotting (Figure 5A). According to the outcomes, it was noticeable that the use of FGF1 resulted in higher expression levels of the upstream proteins PI3K, p38 and p-p38 in the treated endometrium than in the thin endometrium (Figures 5B–D). The expression levels of autophagy indicator protein LC3II and beclin-1 protein also changed accordingly, with trends consistent with those of the upstream pathway proteins (Figures 5F, G). The SQSTM1/p62 is a key protein in autophagy. The normal expression levels of SQSTM1/p62 were decreased after autophagy activation. However, in the present experiment, SQSTM1/p62 expression was increased after FGF1 therapy (Figure 5E).

For further explanation of the activation of the autophagic pathway as a result of FGF1, 3-MA (10 mg/kg), an inhibitor of autophagy, was intraperitoneally injected into rats for 1 week after drug administration. The tissues were taken again to assess the changes in expression of autophagy-related proteins (Figure 5H). 3-MA suppressed the FGF1-induced increase in PI3K, p38, p-p38, SQSTM1/p62, beclin-1 and LC3Ⅱ (Figures 5I–N).

## Discussion

The thickness of the endometrium is one of the key factors in the successful implantation of an embryo by *in vitro* fertilization (Hosseini Aghdam et al., 2022; Zheng et al., 2022). The common clinical causes of thin endometrium are reduced VEGF production and decreased estrogen concentration, and numerous treatments have been proposed to address these causes; however, the results are not as good as they could be (Xie et al., 2020). The treatment of thin endometrium remains a challenge in reproductive medicine. While FGF1 has been studied in the past for its role in fat metabolism and diabetic wounds (Wang et al., 2020a; Deptula et al., 2021), its use in skin repair has also been reported in recent years (He et al., 2019). In addition, Guan et al (Guan et al., 2022) found that FGF1 combined with hydrogel could be used to treat intrauterine adhesions. On this basis, we hypothesized that FGF1 could promote endometrial thickening and facilitate recovery of endometrial function.

In the present study, a thin-layered endometrial rat model was successfully constructed using ethanol, the therapeutic effect of FGF1 on thin endometrium was determined and its mechanism of effect was explored. According to the results, FGF1 promoted the proliferation of endometrial epithelial and mesenchymal cells, as well as blood vessel formation, thereby thickening the endometrium and providing a better environment for embryo implantation. Additionally, FGF1 mediated cellular and vascular facilitation by enhancing the PI3K/p38/beclin-1 autophagic pathway.

Observations of the shape of the uterus and the results of H&E staining revealed that the restorative effect of FGF1 on the uterus was not only reflected in changes in the thickness of the endometrium but also in the external morphology of the uterus. FGF1 was able to improve the uterine stenosis and bruising associated with ethanol injury and restored the uterus to its original state. In addition, a reduction in the number of endometrial cells, blood vessels and glands, as well as loss of epithelial cells in the damaged group, were observed. However, in the FGF1 group, all these conditions were improved, especially the numbers of glands and cells.

PCNA is a protein marker that indicates the number increase of cells. Vim is a type III intermediate filament protein with several basic cellular functions, including cell migration, proliferation and division. It is often used to observe the epithelial-mesenchymal transition in various diseases, especially cancer (Fang et al., 2022; Holmberg et al., 2022; Ma et al., 2022). In the present study, vim and PCNA were used as indicators of endothelial mesenchymal cell proliferation. Based on the experimental results, the effect of FGF1 on the endometrium was mainly focused on promoting the proliferation of endometrial mesenchymal cells. Although vWF, a marker of vascular proliferation, also exhibited changes in expression in the treated and injured groups, the extent of this change was less pronounced than that of PCNA and vim. Immunohistochemistry analysis of CK19 and MUC-1 also demonstrated the same results as aforementioned.

Endometrial fibrosis has been reported in the past as a key component of endometrial injury (Liu et al., 2019). The main cause of endometrial fibrosis is now considered to be abnormal migration and proliferation of uterine epithelial and mesenchymal cells, leading to abnormal secretion of extracellular matrix proteins and cytokines, resulting in the deposition of type I collagen in the endometrium to form endometrial fibrosis (Hu et al., 2018; Cao et al., 2020). In the present study, FGF1 inhibited endometrial fibrosis, a result that was consistent with the earlier finding of Guan *et al* (Guan et al., 2022) that hydrogel-bound FGF1 suppressed the process of intrauterine endofibrosis.

The main purpose of treating thin endometrium clinically is to enable embryo implantation and growth, and thus, the recovery of endometrial function is crucial. ER expression in the endometrium has a marked impact on the normal female biological cycle and late pregnancy. Abnormalities in its expression can cause a variety of diseases (Yu et al., 2022). Additionally, the combination of progesterone and PR also serves an important role during pregnancy. Immunohistochemistry analysis of ER and PR demonstrated that FGF1 could restore the function of the endometrium.

Recent studies have suggested that the autophagic pathway has a major impact on endometrial thickening. A clinical study used intracavitary physiotherapy combined with acupuncture to improve tolerance in patients with thin endometrium. Analysis of the endometrial tissue of patients revealed that activation of the autophagic pathway mediated the thickening of the endometrium (Qi et al., 2022). Feng *et al* (Feng et al., 2017) revealed that tamoxifen, a drug commonly used in breast cancer, stimulated nuclear factor E2-related factor 2-dependent SQSTM1 transcription and promoted endometrial hyperplasia *via* activation of protein kinase Cδ. Investigating whether inhibition of SQSTM1 or more autophagic markers can reverse the effects induced by triamcinolone acetonide provides more definitive evidence for the potential direct involvement of autophagy in endometrial proliferation. Another experiment clarified that two drugs, metformin and sorafenib, alleviated endometrial hyperplasia in patients with polycystic ovary syndrome by enhancing apoptosis and regulating autophagy. Wang *et al* (Wang et al., 2020b) demonstrated that endometrial cells treated with metformin or sorafenib exhibited upregulated LC3-II expression and downregulated SQSTM1/p62 expression. In previous studies, FGF1 has been demonstrated to correlate with autophagy, and thus, improve spinal cord injury (Li et al., 2018; Xu et al., 2020). Therefore, we hypothesized that FGF1 cures thin endometrium by activating the autophagy pathway. According to the findings of the present study, we hypothesized that FGF1 acting on FGF receptor in the endometrium upregulated the expression of PI3K and p38, thereby activating the autophagic pathway *via* the p38 pathway to promote the proliferation of endometrial cells. The expression levels of LC3-II and beclin-1 in the autophagy pathway naturally increased after FGF1 application. This was consistent with the previously explored relationship between the p38 pathway and the autophagy pathway (Huang et al., 2021; Zhao et al., 2022). However, when comparing our results to those of previous studies, it must be pointed out that SQSTM1/p62 expression was higher after FGF1 activating the autophagy pathway. The speculation for this special phenomenon is that FGF1 promotes the initiation of autophagy leading to a compensatory increase in SQSTM1/p62 (Geffken et al., 2022). Another speculation is that during the action of FGF1, a different pathway is activated, leading to an increase in the expression of SQSTM1/p62 and thus promoting endometrial thickening (Feng et al., 2017; Lu et al., 2022). But the details need to be explored in subsequent studies.

## Conclusion

In the present study, FGF1 was successfully used to treat thin endometrium. Compared with the injured group, FGF1 could increase endometrial thickness in rats by increasing the number of stromal cells and glands, reducing the fibrous area and promoting angiogenesis. By contrast, the proliferative effect of FGF1 on cells was derived from the activation of the PI3K/p38/beclin-1 signaling pathway. As a result, the present study provided a theoretical and experimental basis for the use of FGF1 in the clinical treatment of thin endometrium.

## Data Availability

The raw data supporting the conclusion of this article will be made available by the authors, without undue reservation.

## Abbreviations

FGF1, fibroblast growth factor 1; FGFs, fibroblast growth factor superfamily; 3-MA, 3-methyladenine; BSA, bovine serum albumin; α-SMA, α-smooth muscle actin; MUC-1, mucin 1; ER, estrogen receptor; VEGF, vascular endothelial growth factor; SDS, sodium dodecyl sulfonate; PVDF, polyvinylidene fluoride; PR, progesterone receptor; CK19, cytokeratin 19; PCNA, proliferating cell nuclear antigen; vim, vimentin; vWF, von Willebrand factor; PI3K, phosphoinositide 3-kinase; p-p38, phosphorylated p38; SQSTM1/p62, sequestosome 1/p62; LC3, MAP1LC3.
